# Acute Expressive Aphasia and Confusion As the Initial Neurologic Manifestation of Herpes Simplex Virus Type 1 Encephalitis

**DOI:** 10.7759/cureus.111387

**Published:** 2026-06-23

**Authors:** Veronika Espinal, Antonio Santana, Kumaraswamy Nutalapati, Muhammad Nawaz, Sana Khan

**Affiliations:** 1 Internal Medicine, HCA Florida Sarasota Doctors Hospital, Sarasota, USA

**Keywords:** acyclovir, aphasia, cerebrospinal fluid polymerase chain reaction, encephalitis, herpes simplex virus encephalitis, herpes simplex virus type 1, hyponatremia, temporal lobe

## Abstract

Herpes simplex virus type 1 encephalitis is a neurologic emergency that can present with focal language deficits before the diagnosis becomes clinically apparent. We present an 82-year-old man with diabetes mellitus, hypertension, and hyperlipidemia who developed acute confusion and expressive aphasia after an emergency department visit the previous day for fever, chills, fatigue, sore throat, dizziness, and abdominal discomfort. On arrival for the aphasia presentation, the temperature was 100.3°F, and there were no motor or sensory deficits. Computed tomography of the head, computed tomography angiography of the head and neck, and computed tomography perfusion showed no hemorrhage, large vessel occlusion, or perfusion abnormality. Magnetic resonance imaging of the brain on day 2 showed a focal left temporal lobe abnormality with fluid-attenuated inversion recovery and gradient recalled echo signal change and slight restricted diffusion, without abnormal enhancement. Empiric intravenous acyclovir and broad-spectrum antibiotics were started after magnetic resonance imaging raised concern for encephalitis. Cerebrospinal fluid showed white blood cells 159 cells/µL, 100% lymphocytes, protein 218 mg/dL, glucose 63 mg/dL, and herpes simplex virus type 1 polymerase chain reaction positivity. Electroencephalogram showed diffuse slowing with independent bitemporal sharp waves, at times periodic, without electrographic seizures. His course was complicated by hypotonic hyponatremia, concerning for syndrome of inappropriate antidiuretic hormone secretion, although urine osmolality and urine sodium did not result. Herpes simplex virus type 1 encephalitis should be considered early in older adults with acute aphasia, confusion, recent febrile symptoms, negative vascular imaging, and temporal lobe magnetic resonance imaging abnormalities.

## Introduction

Herpes simplex virus type 1 (HSV-1) encephalitis is the most common sporadic viral encephalitis in adults and remains associated with substantial neurologic morbidity despite antiviral therapy [1,2]. Typical features include fever, altered mental status, seizures, and focal neurologic deficits. However, early presentations may be focal, especially when the dominant temporal lobe is involved.

Acute aphasia often triggers stroke evaluation because dominant middle cerebral artery ischemia can produce language impairment. However, isolated aphasia without motor or sensory deficits may also occur with stroke mimics, including seizure, infection, toxic-metabolic encephalopathy, migraine, and encephalitis [3]. This distinction matters because urgent stroke workup should proceed quickly, while confusion, recent febrile symptoms, and negative vascular imaging should also prompt early consideration of encephalitis.

We present an older adult with acute expressive aphasia and confusion as the initial neurologic manifestation of HSV-1 encephalitis. This case highlights the importance of parallel stroke and encephalitis evaluation when acute aphasia is accompanied by encephalopathy or recent infectious symptoms.

## Case presentation

An 82-year-old man with diabetes mellitus, hypertension, and hyperlipidemia was brought to the emergency department by his wife for abrupt confusion and inability to form coherent sentences. Symptoms began approximately 30 minutes before arrival. He had been evaluated in the emergency department the previous day for fever, chills, fatigue, sore throat, dizziness, and abdominal discomfort. The initial workup at that visit was unrevealing, and he was discharged on amoxicillin for presumed streptococcal pharyngitis.

On arrival for the aphasia presentation, temperature was 100.3°F, blood pressure was 192/88 mm Hg, heart rate was 87 beats/minute, respiratory rate was 16 breaths/minute, and oxygen saturation was 96% on room air. Serum glucose was 274 mg/dL. There were no gross motor or sensory deficits. Speech was confused and later evolved into prominent expressive aphasia. The initial computed tomography (CT) head showed no acute intracranial abnormality. Coronavirus disease 2019 and influenza testing were negative. He was admitted for an altered mental status.

Several hours after admission, an inpatient stroke alert was activated due to worsening expressive aphasia and dysarthria. The National Institutes of Health Stroke Scale (NIHSS) score was 3 for severe aphasia and mild-to-moderate dysarthria. Repeat CT head showed no acute intracranial process. CT angiography (CTA) of the head and neck and CT perfusion (CTP) showed carotid calcified atherosclerosis without significant stenosis, no large vessel occlusion, and no perfusion abnormality. Teleneurology initially favored a small left middle cerebral artery cortical stroke and recommended aspirin, clopidogrel loading, permissive hypertension, telemetry, echocardiogram, and brain magnetic resonance imaging (MRI).

Brain MRI without contrast performed on day 2 showed no definite acute infarct but demonstrated a left temporal lobe signal abnormality with slight restricted diffusion and mild gradient recalled echo (GRE) and fluid-attenuated inversion recovery (FLAIR) signal change (Figure 1).

**Figure 1:**
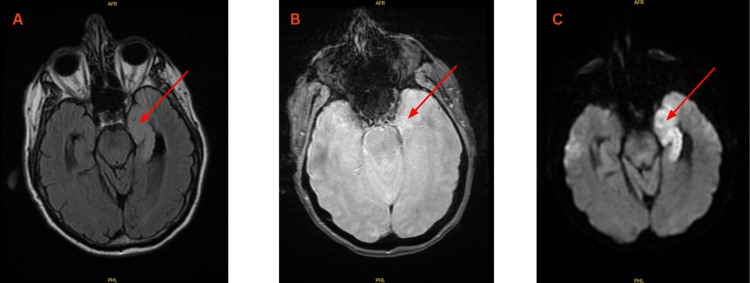
Brain MRI without contrast showing a left temporal lobe abnormality Axial MRI brain without contrast demonstrating a left temporal lobe abnormality. A: Axial T2 FLAIR image with arrow showing left temporal lobe FLAIR signal abnormality. B: Axial T2 GRE image with arrow showing corresponding mild GRE signal change in the left temporal region. C: Axial diffusion-weighted imaging B-1000 image with arrow showing corresponding left temporal restricted diffusion. DWI, diffusion-weighted imaging; FLAIR, fluid-attenuated inversion recovery; GRE, gradient recalled echo

Contrast-enhanced MRI showed the same focal left temporal abnormality without abnormal enhancement, with differential diagnosis, including encephalitis or infiltrative process (Figure 2).

**Figure 2:**
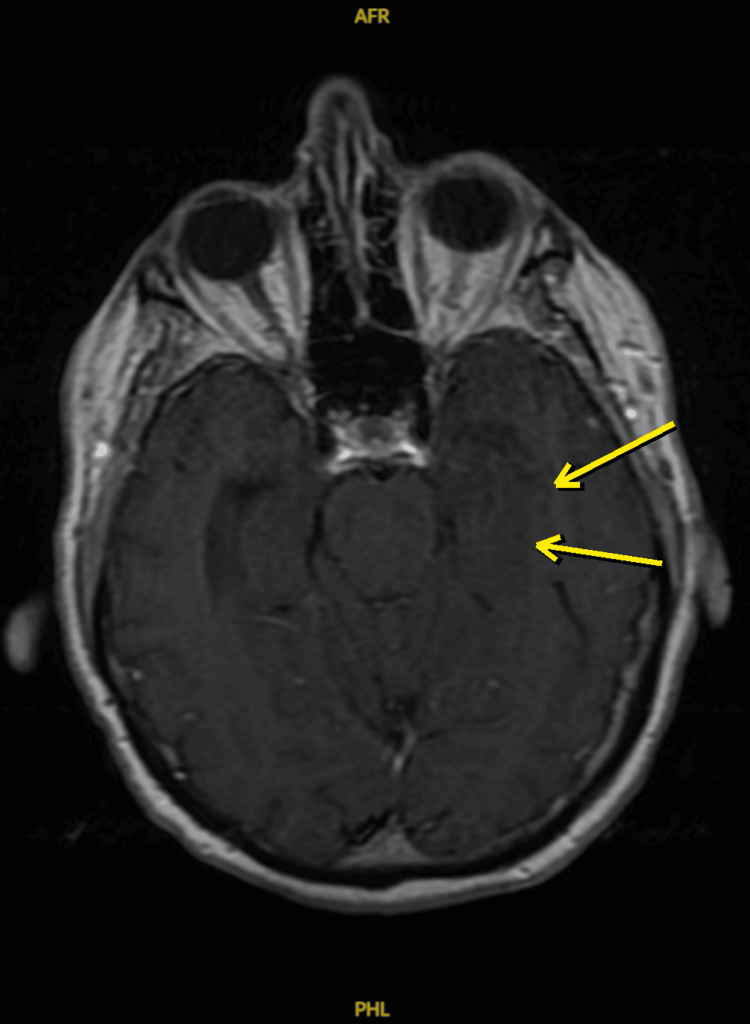
Contrast-enhanced brain MRI showing a nonenhancing left temporal lobe abnormality Contrast-enhanced axial brain MRI demonstrating a focal left temporal lobe abnormality without abnormal enhancement. Arrows indicate the left temporal lobe region corresponding to the abnormality seen on noncontrast MRI sequences.

Given the recent febrile symptoms, confusion, expressive aphasia, negative vascular imaging, and temporal lobe MRI abnormality, empiric intravenous acyclovir and broad-spectrum antibiotics were started on day 2 after the MRI raised concern for encephalitis. Lumbar puncture was ordered. Ideally, cerebrospinal fluid (CSF) sampling should be obtained before antimicrobial therapy when it can be performed safely and without delaying treatment.

Lumbar puncture showed xanthochromic CSF with white blood cells 159 cells/µL, 100% lymphocytes, protein 218 mg/dL, glucose 63 mg/dL, and HSV-1 polymerase chain reaction (PCR) positivity. Herpes simplex virus type 2 PCR and cryptococcal antigen were negative. Broad-spectrum antibiotics were discontinued after HSV-1 encephalitis was confirmed and bacterial meningitis was considered less likely. Pertinent laboratory and CSF findings are summarized in Table 1.

**Table 1:** Pertinent laboratory and cerebrospinal fluid findings CSF, cerebrospinal fluid; HSV-1, herpes simplex virus type 1; HSV-2, herpes simplex virus type 2; PCR, polymerase chain reaction; SIADH, syndrome of inappropriate antidiuretic hormone secretion

Test	Result	Reference range	Interpretation
Serum glucose on arrival	274 mg/dL	70-99 mg/dL	Hyperglycemia
Serum sodium on admission	130 mmol/L	136-145 mmol/L	Mild hyponatremia
Serum sodium nadir	126 mmol/L	136-145 mmol/L	Worsening hyponatremia
Serum sodium at discharge	133 mmol/L	136-145 mmol/L	Improved by discharge
Serum osmolality	270-283 mOsm/kg	275-295 mOsm/kg	Initially hypotonic
Urine osmolality	Not resulted	Variable	SIADH could not be confirmed
Urine sodium	Not resulted	Variable	SIADH could not be confirmed
CSF white blood cells	159 cells/µL	0-5 cells/µL	Pleocytosis
CSF lymphocytes	100%	Variable	Viral or inflammatory pattern
CSF protein	218 mg/dL	15-45 mg/dL	Elevated
CSF glucose	63 mg/dL	40-70 mg/dL	Preserved glucose
CSF HSV-1 PCR	Positive	Negative	Diagnostic
CSF HSV-2 PCR	Negative	Negative	HSV-2 not detected
CSF cryptococcal antigen	Negative	Negative	Cryptococcal meningitis less likely

An electroencephalogram (EEG) was obtained to evaluate for seizure activity. It showed moderate diffuse background slowing with independent bitemporal sharp waves, at times periodic, without electrographic seizures. The EEG interpretation noted that this pattern was compatible with herpes encephalitis in the appropriate clinical setting.

The patient’s serum sodium decreased from 130 mmol/L on admission to 126 mmol/L with low serum osmolality. Hyponatremia was concerning for syndrome of inappropriate antidiuretic hormone secretion (SIADH) in the setting of HSV-1 encephalitis, but SIADH could not be confirmed because urine osmolality and urine sodium were unavailable. Hypovolemic hyponatremia could not be fully excluded. He was treated with fluid restriction and urea sodium 15 g twice daily, with serum sodium improving to 133 mmol/L by discharge.

Renal function was monitored during acyclovir therapy. Serum creatinine increased from 0.76 mg/dL to 1.04 mg/dL by discharge. Infectious disease recommended intravenous acyclovir 10 mg/kg every eight hours for a planned 21-day course, with close renal monitoring and intravenous fluids to reduce nephrotoxicity risk.

He had persistent expressive aphasia but no clinical or electrographic seizures. He was discharged to a skilled nursing facility for completion of intravenous acyclovir and rehabilitation.

## Discussion

This case illustrates a practical diagnostic challenge in the evaluation of acute aphasia. The initial stroke evaluation was appropriate because abrupt expressive aphasia can reflect dominant hemisphere ischemia. However, recent febrile symptoms, confusion, absence of large vessel occlusion or perfusion abnormality, and subsequent left temporal lobe MRI abnormality supported early consideration of encephalitis. In this setting, stroke evaluation and empiric encephalitis treatment should proceed in parallel rather than sequentially.

The temporal lobe MRI abnormality was the key radiologic finding. HSV-1 encephalitis classically involves the temporal and frontal lobes, and MRI is the preferred imaging modality when encephalitis is suspected [1,2]. In this patient, MRI showed left temporal FLAIR and GRE signal abnormality with slight restricted diffusion and no abnormal enhancement. Lack of enhancement did not exclude encephalitis because enhancement may vary by timing and severity.

This case also illustrates why acute aphasia should not be attributed to stroke alone when encephalopathic or infectious features are present. Casella et al. reported that isolated aphasia in the emergency department was more often due to stroke mimic than infarction, although true ischemic stroke remains possible and requires urgent evaluation [3]. Prior HSV encephalitis cases have described similar aphasia-dominant presentations [4-6]. The present case adds the combination of acute expressive aphasia and confusion as the initial neurologic manifestation, recent febrile symptoms, negative CT, CTA, and CTP, nonenhancing left temporal MRI abnormality, HSV-1 PCR confirmation, hypotonic hyponatremia, and bitemporal epileptiform discharges without overt seizures.

CSF testing confirmed the diagnosis. The patient had lymphocytic pleocytosis, elevated protein, preserved glucose, and HSV-1 PCR positivity. Infectious Diseases Society of America guidelines recommend CSF analysis with HSV PCR testing in suspected encephalitis and recommend empiric acyclovir in all patients with suspected encephalitis while diagnostic studies are pending [1]. In this case, empiric acyclovir and broad-spectrum antibiotics were started on day 2 after an MRI suggested encephalitis. Once HSV-1 PCR returned positive and bacterial meningitis was considered less likely, antibiotics were discontinued, and acyclovir was continued.

The hyponatremia is a useful secondary clue, but should be interpreted cautiously. Hyponatremia has been reported more frequently in HSV-1 encephalitis than in other viral encephalitides. Basaran et al. found hyponatremia, defined as serum sodium less than 135 mmol/L, in 56.3% of HSV-1 encephalitis cases compared with 19.6% of non-HSV-1 viral encephalitis cases [7]. In this patient, hypotonic hyponatremia was concerning for SIADH, but urine osmolality and urine sodium were not obtained. Therefore, the most accurate interpretation is hypotonic hyponatremia concerning for SIADH rather than confirmed SIADH.

EEG was supportive but not diagnostic. EEG in HSV encephalitis may show focal slowing, epileptiform discharges, seizures, or periodic discharges, often involving temporal regions [8]. In this patient, the EEG was mainly useful to evaluate for nonconvulsive seizure activity and showed no electrographic seizures. The diagnosis was established by CSF HSV-1 PCR.

Early recognition matters because delayed acyclovir is associated with worse outcomes, and HSV encephalitis can cause persistent neurologic disability despite treatment [1,2]. This patient was discharged with persistent expressive aphasia, underscoring the morbidity of HSV-1 encephalitis and the importance of considering empiric antiviral therapy when acute aphasia is accompanied by confusion, recent febrile symptoms, and temporal lobe abnormalities.

## Conclusions

HSV-1 encephalitis can present early with acute expressive aphasia and confusion, especially when the dominant temporal lobe is involved. In older adults with aphasia, encephalopathy, recent febrile symptoms, negative vascular imaging, and temporal lobe MRI abnormalities, clinicians should consider encephalitis early, obtain CSF studies when safe, and start empiric intravenous acyclovir without unnecessary delay. Hypotonic hyponatremia and temporal EEG abnormalities may support the diagnosis, but CSF HSV PCR remains central.
